# Generative Design in Building Information Modelling (BIM): Approaches and Requirements

**DOI:** 10.3390/s21165439

**Published:** 2021-08-12

**Authors:** Wei Ma, Xiangyu Wang, Jun Wang, Xiaolei Xiang, Junbo Sun

**Affiliations:** 1 School of Design and the Built Environment, Curtin University, Bentley, WA 6102, Australia; wei.ma@curtin.edu.au (W.M.); xiaolei.xiang@curtin.edu.au (X.X.); tunneltc@gmail.com (J.S.); 2 School of Civil Engineering and Architecture, East China Jiao Tong University, Nanchang 330013, China; 3 Australasian Joint Research Centre for Building Information Modelling, School of Built Environment, Curtin University, Perth, WA 6102, Australia; 4 School of Engineering, Design and Built Environment, Western Sydney University, Kingswood, NSW 2747, Australia; jun.wang@westernsydney.edu.au

**Keywords:** generative design, building information modelling, technology integration, methodological relationships, skill requirement and improvement, novel development perspectives

## Abstract

The integration of generative design (GD) and building information modelling (BIM), as a new technology consolidation, can facilitate the constructability of GD’s automatic design solutions, while improving BIM’s capability in the early design phase. Thus, there has been an increasing interest to study GD-BIM, with current focuses mainly on exploring applications and investigating tools. However, there are a lack of studies regarding methodological relationships and skill requirement based on different development objectives or GD properties; thus, the threshold of developing GD-BIM still seems high. This study conducts a critical review of current approaches for developing GD in BIM, and analyses methodological relationships, skill requirements, and improvement of GD-BIM development. Accordingly, novel perspectives of objective-oriented, GD component-based, and skill-driven GD-BIM development as well as reference guides are proposed. Finally, future research directions, challenges, and potential solutions are discussed. This research aims to guide designers in the building industry to properly determine approaches for developing GD-BIM and inspire researchers’ future studies.

## 1. Introduction

Generative design (GD) as a rule-driven iterative design process is based on algorithmic and parametric modelling to automatically explore, iterate, and optimise design possibilities by defining high-level constraints and goals [1,2]. Building Information Modelling (BIM) is a collection of regulations, procedures, and technologies enabling the creation, recording, and management of buildings’ digital information through their entire life cycle [3,4,5,6,7]. The GD-BIM integration combines a new intelligent design approach and the technology of automated construction information generation [8,9,10,11]. It can facilitate the constructability of GD’s automatic design solutions, and meanwhile improve BIM’s capability in the early design phase. Thus, developing GD-BIM has drawn increasing attention academically and practically [12,13,14,15,16].

The current research regarding GD-BIM development mainly focuses on exploring applications and investigating tools. For instance, some GD-BIM are developed and studied to creatively tackle design issues, while some research examine software and programming means to compare developing tools [17,18,19,20,21]. However, there are a lack of methodological relationship studies, specifically, determination of proper developing approaches, skill requirements, and improvement paths based on different development objectives or GD properties; thus, the threshold of developing GD-BIM still seems high. It is especially difficult for those designers in the building industry with little knowledge of GD or programming. Therefore, appropriate and practicable methodological guidance is in great demand to provide designers advice on developing GD in BIM. 

The aim of this review is to investigate the current approaches of developing GD in BIM to discover methodological relationships, skill requirements, and improvement, to support designers on proper method selection for developing GD-BIM. Thus, publications regarding developing GD in BIM over the last decade are searched and reviewed in this study. Section 2 clarifies the necessity and logic of developing GD in BIM by investigating background knowledge. Section 3 explains research methods. Section 4 reviews, compares, and analyses the objectives, programming language suitability, and skill learning of developing GD-BIM, to propose perspectives of objective-oriented, GD component-based, and skill-driven GD-BIM development. In this section, a set of reference guides are suggested to designers on development methods selection, skill learning, and improving paths. Section 5 discusses future research directions and challenges and recommends potential solutions. 

## 2. Generative Design and Building Information Modelling 

This section aims to clarify the necessity and logic of developing GD in BIM by investigating background knowledge of GD, GD components, BIM, and GD-BIM integration.

### 2.1. Generative Design

GD is a rules-driven iterative design process [1]. It is based on algorithmic and parametric modelling to automatically explore, iterate, and optimise design possibilities by defining high-level constraints and goals [2]. Shea et al. [22] stated the aim of GD is to ‘‘explore creative and constructable designs by creating new design processes using the latest computing and manufacturing capabilities’’. With development of computer power and growing interest of researchers and practitioners, GD has become a new intelligent design approach and has been studied and applied in many fields academically and practically [8,23,24,25,26,27,28,29,30,31,32,33,34,35,36,37]. 

The most powerful capability of GD is to automatically explore and iterate design possibilities, and permutate the best solutions to human designers for decision-making [33]. This process usually occurs at the conceptual design stage, where GD can operate while other CAD applications are unable to support [38]. In fact, GD can explore design possibilities for any type of AEC designs (e.g., architectural design, structural design, interior design, urban design, or urban planning, etc.) at the design formulation stage. Thus, it is considerably useful to implement GD at the early design stage in the AEC industry.

### 2.2. Components of a GD

Currently, there are different classifications of GD components from various perspectives. Krish [38] breaks down the GD process into three components from the perspective of design: (1) “a design schema”, (2) “a means of creating variations”, and (3) “a means of selecting desirable outcomes”. Marsh [39] discussed GD components from the view of performance measurement: (1) “Configuration Variation”, (2) “Performance Metric”, and (3) “Decision-Making Response”. Nagy and Villaggi [2] states from the angle of formulating GD: (1) “a generative model expressing broad design possibilities”, (2) “an evaluative component consisting of proposed design targets”, and (3) “a metaheuristic search algorithm navigating the design iterations”. It is seen that the classifications are determined based on the corresponding research objectives. Thus, in this study, a GD will be decomposed from the aspect of development, to facilitate the investigation of relationships between GD properties and development methods.

Generally, creating a GD consists of several steps: define design goals, formulate design constraints, determine algorithms, program the GD, run the GD, and modify the generated parametric models based on goals and constraints until satisfied [32,38]. Among them, programming the algorithms and design constraints are the major development process, as the design goals are pre-defined, while the parametric models are automatically generated accordingly [2]. Therefore, in this study, a GD is decomposed into two major components from the perspective of developing its: (1) algorithm, and (2) design constraints. 

The algorithm is usually highly abstract as it is used to instruct the computer to generate and optimise design possibilities through search methodologies. Singh and Gu [33] summarised five commonly used algorithms, namely, generative design techniques, as: Genetic Algorithms, Shape Grammars, L-systems, Swarm Intelligence, and Cellular Automata. As for usages, the proper selection of algorithms is determined according to the characteristic of design objectives [33]. For instance, if the design purposes are to discover space layout or visual compositions exploratorily, then the Shape Grammars algorithm is most likely selected. Or, if the objective is design improvement or optimisation, then the Genetic Algorithm is usually employed.

The design constraints are relatively intuitive and used to limit the scope of design exploration and narrow the search range of optimisation. The design constraints are a series of design conditions or preferences, such as spatial or morphological requirements, dimensional constraints, materials selections, manufacturing methods, or even cost constraints, etc. [25,28,40]. They are scripted by designers in a computer recognizable format to control the design exploration, iteration, or evolution [41,42,43]. As for scripting, design constraints are mostly manual scripted; however, research has started to develop automatic methods. For example, approaches to extract constraints-related information from text format to computer recognisable format have been studied and developed [44,45,46,47]. Yet, manually scripting of design constraints is still more common and flexible so far.

To identify the methodological relationship between development methods and the GD components, their different characteristics and properties need to be further studied. Detailed analysis is elaborated in Section 4 (Analysis section). 

**Figure 1:**
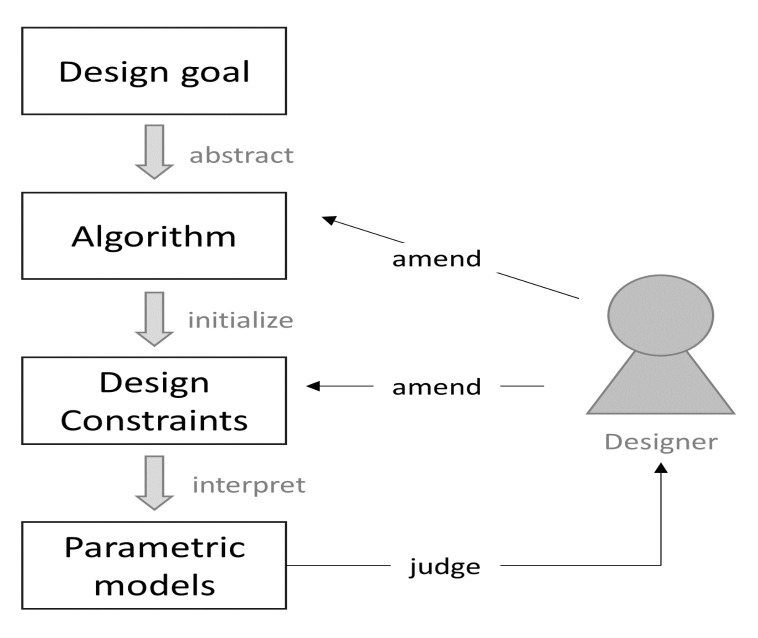
Relationship of GD components and the process of running a GD.

A figure is proposed to indicate the relationship of GD components and the process of running a GD, as presented in Figure 1. Firstly, the design goal is inputted into the GD program in the computer along with appropriate algorithms and well-defined design constraints. Then, parametric models are generated, iterated, and permuted to designers for decision-making. Finally, designers modify parameters in the algorithm and design constraints to adjust models until design goals are achieved.

### 2.3. Building Information Modelling

As a revolutionary technology, BIM has rapidly changed the paradigm of a building’s conception, design, construction, and operation [48,49,50,51]. Back to 1970s, the initial research of BIM began as parametric modelling research; however, the practical implementation of BIM in the building industry started from the mid-2000s [48]. Since then, BIM has quickly become the centrepiece of AEC technology [48]. Wang et al. [3] demonstrate that BIM is a collection of regulations, procedures, and technologies enabling interacting to create a “digital representation of the projects’ physical and functional characters”. In BIM, digital formatting of the building’s fundamental design and information data can be recorded and managed through the projects’ entire life cycle [4,5,6,7]. Meanwhile, the quality of designs and datasets in BIM can be well controlled and improved by various approaches [52,53]. Therefore, it enables manipulation and maintenance of shared data and information resource for all users [54,55,56]. In conclusion, “BIM is not just software; It is a process and software” [48]. 

BIM software (e.g., Revit or ArchiCAD, etc.) provide an Application Programming Interface (API), allowing the access, extraction, selection, and modification of a building’s data and information. Besides, the API provides users a platform to develop add-ons by writing a program or script to extend the application’s capabilities [57,58]. For instance, the Revit API enables proficient Revit users to customize their own tools by programming with it to enhance Revit’s capability, and to improve workflows [58]. 

### 2.4. Integration of GD with BIM 

Although BIM is applicable throughout the project’s entire life cycle, its usages in the design phase are mainly limited in the later design stages (e.g., the design development stage, structural design stage, mechanical, electrical, and plumbing design stage, etc.). In terms of design exploration in the early design stage or the creative phase (e.g., the conceptual design stage), the capability of BIM is inadequate. However, integrating GD demonstrates the potential to make up this deficiency of BIM. 

GD, as a computer-aided design (CAD) method, mostly focuses on geometrical modelling [38,59] to quickly explore design. However, the “information” attribute usually cannot be generated. Differently, BIM produces components with both geometry and information attributes to facilitate buildability [59,60,61,62]. Thus, integrating BIM can improve the constructability of design solutions generated by GD. 

The GD-BIM integration is beneficial to improve each other’s capability by making up mutual deficiencies. The integration can support automatic and fast design explorations and enable buildability of these GD solutions, while extending BIM’s capability in the early design phase. 

To implement the integration, the API in BIM provides possibility and platforms. In fact, GD as a new feature is already available in Revit 2021 [63], the latest Revit version. Although the current GD availability in Revit is limited (only three design studies), the exploration and customization of more GD is allowed by using the Revit API. Therefore, developing GD in BIM (e.g., Revit) has a promising future.

## 3. Review Methodology

This research conducted a critical review using the content analysis-based review method [64,65,66,67] to obtain a novel understanding of methodological relationships, skill requirements, and improvement for developing GD in BIM. Scopus is selected as the searching database in this study as it is claimed the “largest abstract and citation database” [68] and provides the broadest overview of international and interdisciplinary scientific data and literature [69]. “Generative design” and “building information modelling” or “BIM” are the primary keywords for searching. However, GD is often confused with parametric design (PD) and algorithm design (AD), and used in parallel [70,71,72,73]. Therefore, it is imperative to understand the relationship among these items before determining the final searching keywords. According to Caetano et al. [70], “AD is a subset of GD”, and “PD is orthogonal to AD and GD”, which means AD and certain PD can be considered as GD as well. Therefore, AD and PD are added as a search key word as well to make the literature search as comprehensive as possible and avoid missing literature. To conclude, the keywords are determined as “generative design” OR “parametric design” OR “algorithm design” AND “building information modelling” OR “BIM”.

The past 10 years (2010–2020) was set as the time frame for searching because developing GD in BIM is a newly emerged research area. There have been great leaps in computational design, including GD and BIM, in the last decade; therefore, review of the past 10 years period can provide a relatively sufficient overview of the present GD-BIM development [28]. The document type was limited to articles and conference papers to ensure the publication quality. 

As such, the search criteria are as following:

- Scopus as the search database.
- Search within: article title, abstract, keywords.
- Search keywords: “Generative design” OR “Parametric design” OR “Algorithm design” AND “Building information modelling” OR “BIM”.
- Publications published between 2010 and 2020.
- Document type: articles and conference papers.
- Publication language: English.

Initially, 114 documents were found in the Scopus database, based on the above search criteria. Non-related subject areas (e.g., Mathematics, Business, Management and accounting, Energy, Medicine, Chemical Engineering, Economics, Econometrics and Finance, Physics and Astronomy) are then excluded. Finally, 93 publications were selected as highly relevant to this study area for review; 61 out of the 93 publications are conference articles (accounting for 66%), while the rest 32 are journal papers (accounting for 34%). 

**Figure 2:**
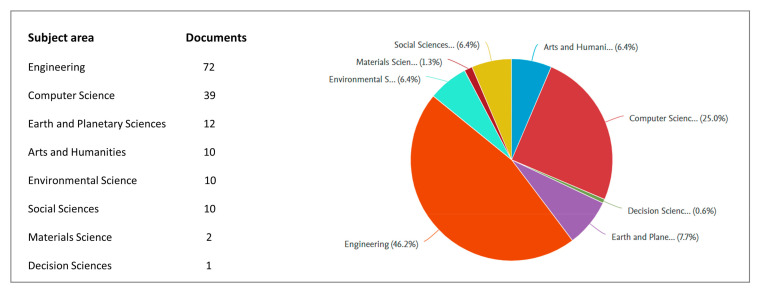
Distribution of subject areas of the selected 93 publications.

The subject area of ‘Engineering’ makes up the largest proportion (46.2%), as shown in Figure 2, which indicates the domain emphasis. Table 1 summarizes the top 5 sources (in terms of amount and year) where the selected publications were published, including The Association for Computer-Aided Architectural Design Research in Asia (CAADRIA), Automation in Construction, IOP Conference Series: Earth and Environmental Science, International Symposium on Automation and Robotics in Construction (ISARC), and Procedia Engineering. Table 1 indicates an increasing interest in this domain study. 

To investigate the methodology of developing GD in BIM, it is essential to understand the development objectives, characteristics of the development objects, development tools, and skill requirement. Accordingly, the categories and characteristics of GD-BIM development objectives, characteristics of GD components, features and applications of programming languages, and skill requirement and improvement frame the detailed review in this study. Based on that, three major topics are analysed to discover the methodological relationships and skill requirements, including:

1. Relationships between programming languages and developing different GD-BIM objectives.
2. Relationships between programming languages and developing different GD components.
3. Skill requirement and learning paths of programming languages for developing GD-BIM.

Mixed methods of quantitative and qualitative research are used to review and analyze the above three topics. Firstly, qualitative research method is used to categorise different objectives of GD-BIM development, followed by quantitative research to investigate relationships between GD-BIM development objectives and applications of programming languages. Secondly, qualitative research is employed to examine the characteristics of GD components and programming languages respectively to analyze the suitability relationship between programming languages and developing GD components. Thirdly, to review discussions on skills of programming languages used to develop GD in BIM from the selected literature and qualitatively study the skill requirements and improvement paths. Accordingly, three novel perspectives of GD-BIM development and a set of reference guides are proposed. Detailed analysis is elaborated in the following section. 

## 4. Analysis

In this section, the GD-BIM development objectives, programming languages suitability, and programming skills learning and improvement are reviewed and analysed to investigate the methodological relationships. Accordingly, perspectives of objective-oriented, GD component-based, and skill-driven GD-BIM development are proposed, as well as a set of reference guides to designers in the building industry. 

### 4.1. Objectives of Developing GD in BIM and the Relationship to Programming Languages

#### 4.1.1. Categorising and Comparison of Different Objectives of GD-BIM Development 

Through analysing the different characteristics of the objectives, the GD-BIM development can be classified into two major categories: (1) solve specific design tasks, and (2) support design processes. Table 2 compares these two categories in detail. The development category of solving specific design tasks is usually project-based and mainly developed by practitioners aiming to solve individual design tasks creatively and efficiently within larger practical projects. To achieve this objective, GD is used to automatically explore and optimise design alternatives, while BIM generates multidimensional information to facilitate the constructability. Detailed information of the examples for this objective category can be found in [74,75,76,77,78,79,80,81], presented in Table 1. The advantages of these GD-BIM developments are targeted and practical problem-solving, lower development difficulty, and a shorter development period. However, the drawbacks of these developments are less universal (as they are conducted based on specific practical design conditions) and too preliminary for complicated tasks.

The development category of supporting design processes is not based on projects; instead, it is mainly developed by researchers, aiming to support certain design processes by establishing environments or systems in the context of BIM. These developments can enhance the applicability, practicality, and compatibility of BIM in certain design stages or fields. For instance, some GD-BIM are created to support designs in certain phases, such as the conceptual design phase, interior design phase, structural design phase, or design customization stage, etc. [57,82,83,84,85,86,87,88,89,90,91,92,93,94,95,96]. Meanwhile, some studies concentrate on improving the portability of GD-BIM developments [59,97,98,99]. These developments have advantages of higher universality, as they can work as platforms or programs allowing use by a wider group of designers for similar design purposes. However, the disadvantages are higher programming requirement, higher developing difficulty (due to the need of multidisciplinary involvement), and long development cycle (due to the need of constant testing and iteration before practical application). Also, most of the current developments are at the prototype stage or the rhetorical stage, and too elementary to support complex design processes [83,87,93].

**Table 2:** A summary of objective categories of developing GD in BIM.

Objective Category	Description	Sub-Objectives	Characteristic	Evaluations	Examples	Programming Method	Programming Language
1. Solve specific design tasks.	This objective is to solve individual design tasks creatively and efficiently within larger practical projects.	Cope with design changes efficiently. Shorten the design period. Generate, document, and fabricate designs in great detail. Improve design efficiency by integration with traditional design workflow. Generate parametric models efficiently. Explore building forms automatically. Explore and generate facade designs automatically.	Project based. Practical design task orientated. Mostly developed by industry practitioners.	Relatively easy to develop and amend. Short development period.Relatively easy programming requirement. Solutions are high targeted but less universal. Currently too preliminary for complicated tasks.	Automate design and production for practical tunnel projects [74].	Grasshopper (in Rhino) and Dynamo (in Revit) used in the Application Programming Interfaces (API’s).	Visual programming language (VPL)
					Digital aided façade design [75].	An add-in named GA (generative design) in Grasshopper.	VPL
					Digital workflows in contemporary architecture and construction [80].	Objected-oriented programming, functional programming, visual programming, and distributed visual programming [80], based on cases.	Textural programming language (TPL) and VPL
					An algorithmic BIM approach in a traditional design studio [81].	Python and AutoLisp are used to script algorithm; Rosetta is used to support BIM back-end.	TPL
					BIM-integration of solar thermal systems [76].	Dynamo in Revit.	VPL
					BIM-based parametric modelling to Tapered Slip-Form System [77].	SmartParts Script Language of Allplan (a BIM tool).	TPL
					Parametric design of Shanghai Tower’s form and façade [78].	Microsoft Visual C# that ran between Grasshopper and Revit [78].	TPL.
					BIM façade module for diagrid twisted structures [79].	Dynamo in Revit.	VPL
2. Support design processes.	This objective aims to support design processes in BIM by building environments or systems integrating with GD in the context of BIM.	Automate the design evaluation. Automate designing, modelling, and documenting of customized designs. Propose an early design workflow in reducing construction waste. Enhance the applicability of BIM in various design stages (such as conceptual design, structural design, etc.). Enhance the practicality of BIM in various types of design (such as interactive brickworks design, green building design, and adaptive facade design, etc.). Establish a portable platform to develop GD in BIM. Customise design tools.	Research based. System development orientated. Mostly developed by researchers.	Higher programming requirement. Higher development difficulty. Long development circle. Solutions are higher universally, but at the prototype stage so far. Currently too elementary to support complex design processes.	Generative design for building interiors using BIM [57].	domain-specific language (DSLs) is used to script the design rules.	TPL
					Generative BIM workspace for conceptual design automation [83].	C#.Net in Revit Add in.	TPL
					Generative interior design using BIM [84].	DSLs to script the design rules.	TPL
					Automatic structural design of RC wall-slab buildings in BIM [85].	Not mentioned.	N/A
					Automated design and modelling for mass-customized timber structure housing [86].	Grasshopper in Rhino.	VPL
					From layout generation to construction document production of customised apartment plans [82].	Existing grasshopper (GH) workflow nodes are used to script design rules, Python in GH is used to create new nodes and script the algorithm, and the processing language is used to develop the Graphic User Interface (GUI).	VPL and TPL
					A novel construction waste reduction workflow using parametric design and module coordination [87].	Existing nodes in GH are used to develop the algorithm.	VPL
					Portable generative design for BIM [59].	An Integrated Development Environment named Rosetta is used to support various TPLs as a front-end, and a series of CAD and BIM applications are connected as back-ends for GD model generation.	TPL
					Design and analysis in a generative tool with multi back-ends [97].	Same as the above.	TPL
					Towards cloud informed robotics [88].	Visual programming for parametric design.	VPL
					A framework for a dimensional customization system [89].	Not mentioned.	
					A Green-BIM approach for adaptive building facade optimisation [90].	Dynamo is used for information extraction; C# is used to develop compliance checking systems.	VPL and TPL
					Virtual generative BIM workspace for maximising conceptual design innovation in the AEC industry [91].	C#.Net programming.	TPL
					Exploit AEC conceptual design innovation by integrating GD with BIM [92].	C#.Net programming	TPL
					G-BIM framework and development process for design automation [93].	C#.Net programming	TPL
					Integrated generative technique for brickworks interactive design [94].	Grasshopper in rhino is used to script the algorithm. Processing is used to create the sketch tool.	VPL and TPL
					Parametric and generative methods with BIM [95].	C#	TPL
					Design of parametric software tools [98]	Grasshopper in Rhino	VPL
					Tool design for architectural design [99].	Grasshopper in Rhino	VPL
					Parametric design based on BIM for sustainable buildings [96].	C# programming in Revit API	TPL

#### 4.1.2. Publications of Different Objectives of GD-BIM Development

**Figure 3:**
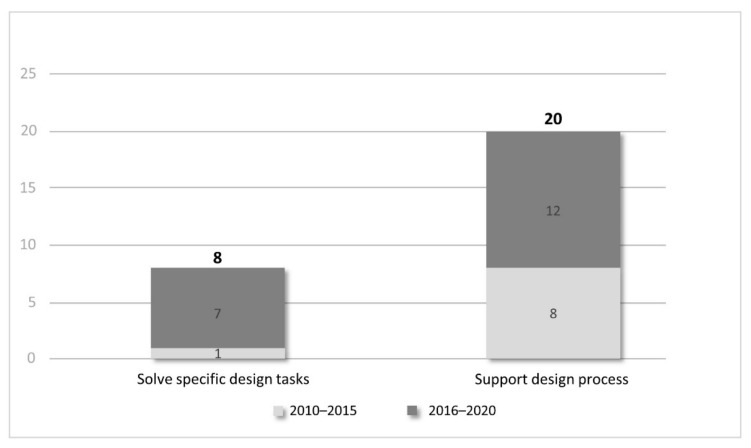
Distribution of publications on different objectives of GD-BIM developments (2010–2020).

Out of the filtered 93, 28 literatures (listed in Table 1) have discussed developing GD in BIM. Figure 3 shows the distribution of publications based on the different objectives classified above. Publications about the objective of “support design processes” occupy the majority (20 in 28), which indicates the research focus and preference in the academic domain. Moreover, the increasing number over the last five years illustrates growing research in this area of study. 

#### 4.1.3. Application of Programming Languages Based on Different Objectives

**Figure 4:**
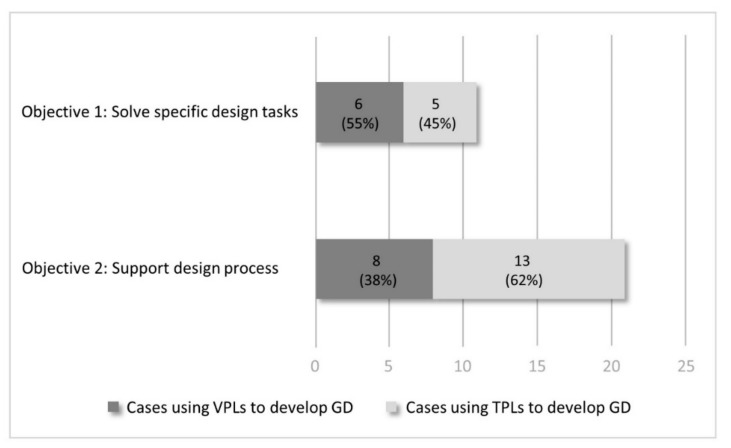
Application distributions of programming languages based on objectives of GD-BIM development.

Programming methods and languages are examined and listed following each example in Table 1. Categories of textural programming languages (TPL) and visual programming languages (VPL) are adopted and investigated in this research. A TPL is a programming language that uses text, code, symbol, and predefined syntax, etc. to develop programs, while a VPL allows users to develop programs by interactively manipulating visual elements, rather than scripting texturally [59,100]. Based on Table 1, there are 6 cases using VPL and 5 cases using TPL in the Objective 1, while 8 cases using VPL and 13 cases using TPL in Objective 2. Accordingly, Figure 4 shows the application distributions of programming languages. It is seen that to develop GD-BIM for solving specific design tasks, the use of VPLs (55%) is slightly more than that of TPLs (45%); to develop GD-BIM for supporting design processes, TPLs are more used than VPLs (62% and 38% respectively). 

Therefore, the methodological relationship can be concluded: VPLs are applied more to individual practical design tasks, for easier and quicker GD-BIM developments, while TPLs are more accepted for harder, longer, and more systematic GD-BIM developments, to establish application platforms or environments. 

#### 4.1.4. Perspective of Objectives-Oriented GD-BIM Development

A novel perspective of objective-oriented GD-BIM development is proposed based on the above analysis, which is the selection of proper development of methods according to the different objectives of developing GD-BIM. Firstly, GD-BIM developments have two major categories based on development objectives, which are “solve specific design tasks” and “support design processes”. Secondly, utilisation of programming methods and languages differ according to different objectives. Thirdly, VPLs are more applicable to easier and quicker GD-BIM development for the objective of solving specific practical design tasks, while TPLs are more capable to develop harder, longer, and more systematic GD-BIM, to establish application platforms or environments for the objective of supporting design processes.

### 4.2. Suitability of Programming Languages for GD-BIM Development

#### 4.2.1. Programming Languages and Software Used to Develop GD in BIM

A programming language and software (usually BIM software) are crucial and indispensable tools in GD-BIM development [17,101,102]. Table 3 compares VPLs and TPLs in detail regarding definitions, languages, advantages, and limitations [12,17,100,101,102,103]. Table 4 illustrates the popular software and applicable programming languages for scripting GD [24,102,104]. As shown in the Table 3, Revit, ArchiCAD, Grasshopper, and GenerativeComponents are the commonly used software for developing GD. Among them, Revit and ArchiCAD are BIM software, while Grasshopper and GenerativeComponents are not (they are CAD software). However, Grasshopper and GenerativeComponents can connect to BIM software by means of plug-ins or a specific edition, as indicated in the Table [101,104]. Therefore, all of them can be considered as platforms for GD-BIM development. As Grasshopper and Dynamo have abundant content in libraries and are easily accessible forums for learners, and Python is easier to learn compared to C++, Grasshopper and Revit are currently more popular for developing GD-BIM.

As indicated in Table 2, TPLs and VPLs have different characteristics, pros, and cons. Based on these features, studies have examined, compared, and evaluated their usages and suitability in GD development [17,59,80,105]. For instance, Leitão et al. [17] have conducted a comparative study to demonstrate that modern TPLs are more productive in developing complex GD, as TPLs have the advantage in abstraction mechanisms. However, due to their difficulty to learn, few designers possess extensive knowledge and proficient ability of TPLs to skilfully use them [59]. In contrast, VPLs are much easier to learn and grasp for beginners because of the advantages of intuitive and interactive properties and simpler operation [106]. However, VPLs’ limitation in sophisticated abstraction mechanism restricts their capability in complex GD development. 

Therefore, in general, VPLs are more suitable to novice designers and simple GD-BIM development, while TPLs are more suitable to expert players and complex GD-BIM development. However, most of the current studies overlook the relationship to GD components, which are inherent properties of GD-BIM. This gap is addressed in the following section.

#### 4.2.2. Suitability Relationship between Programming Languages and GD Component Development

The application of programming languages varies, depending on the properties of GD components. Generally, the algorithms component is highly abstract while the design constraints component is intuitive. Thus, programming languages used to script these components should have the corresponding characteristics. Leitão et al. [17] have summarised the three fundamental dimensions of a full-featured programming language: primitive, combination, and abstraction. Different programming languages have different advantages in these dimensions. Specifically, VPLs have the advantage in primitive dimension, while TPLs have strong points in combination and abstraction dimensions. 

Thus, a suitability relationship is found between programming languages and GD components development, as indicated in Table 5 [12,17,100,103]. VPLs have better primitive dimensions, resulting in good performance of expressing intuitive ideas by simply connecting various primitive components. Thus, VPLs are suitable to develop the “design constraints” component and straightforward algorithms which require intuitive and explicit expression. However, due to the shortcomings in combination and abstraction mechanisms [17], VPLs are not the ideal choice for complex algorithm development (e.g., genetic algorithms, shape grammars, etc.). Due to expression and production in complex and abstract tasks, TPLs are more suitable to develop the complex algorithms component. TPLs can also be used to develop complicated design constraints that require intricate scripting in VPLs. 

Therefore, the methodological relationship can be concluded: TPLs are more suitable to develop abstract components such as the Algorithm component or intricate design constraints, while VPLs are more suitable to develop intuitive components such as the design constraints component or simple algorithms. 

#### 4.2.3. Perspective of GD Component-Based GD-BIM Development

A new perspective of GD component-based GD-BIM development is proposed, which is the selection of proper development methods based on different GD components. Firstly, developing a GD is mainly composed of programming the algorithm and design constraints components. Secondly, the use of programming methods and languages differ based on the different characteristics of the GD components. Thirdly, TPLs are more suitable to develop abstract components, such as the algorithm component or intricate design constraints, while VPLs are more suitable to develop intuitive components such as the design constraints component or simple algorithms.

### 4.3. Programming Skill Learning & Improving for GD-BIM Development

#### 4.3.1. Designers’ Learning of VPLs and TPLs

Research has revealed that VPLs have a faster learning curve, while TPLs have a steeper one [17,100,101,103]. VPLs are easy to learn and use as they were invented to enable easy interaction with computers and became educational tools to teach beginners programming [107]. In terms of conducting design tasks, VPLs enable simpler and more straightforward operation due to their sophisticated IDE and intuitive elements [17]. Thus, VPLs are a good starter for designers to learn programming and create simple programs, despite shortcomings in dealing with complex tasks. 

TPLs are much harder to learn and master because they rely on hard coding and a complicated syntax system, especially for creative professionals such as designers who are not used to linear thinking [103]. However, additional time and effort spent on learning TPLs can be “quickly recovered once the complexity of problems becomes significantly large” [17,80]. Owning knowledge and experience of TPLs can even effectively enhance the productivity of VPL users [17]. Therefore, although hard to learn, TPLs can be a quality weapon for designers to improve design capability and productivity by developing complex programs and customising design tools. 

#### 4.3.2. Influence from Portable Development Environments

Some studies have demonstrated that portable development environments can reduce the requirement of learning new programming skills [17,59,80,81,97,101,108]. For instance, Rosetta as a portable development environment enables a user proficient in one TPL to create GD in various BIM environments without learning extra new programming languages. Rosetta plays as the medium or converter role and allows users to script GD using certain TPLs (as front-end languages) and generate identical GD models in multiple software (as back-end environments). 

Rosetta is beneficial to learning cost and design efficiency because of the portability and interoperability. A study conducted by Caetano and Leitão [81] has demonstrated this, in which Rosetta took advantage of both CAD and BIM to create outstanding design solutions under a very tight deadline. Usually, CAD is more efficient in design exploration than BIM, while BIM is much better at generating construction information. In this project, designers first used a familiar TPL in Rosetta to explore designs and generate design models in a CAD software. Then, once the design was confirmed, designers used the same scripts in Rosetta, but connected to a BIM software to generate an identical model equipped with construction information. Finally, a satisfying design solution with detailed construction guide was produced in a very short time. Hence, learning and use of different programming languages was avoided due to the portability of Rosetta between BIM and CAD, which greatly saved time and improved design efficiency.

Despite the advantage in reducing learning effort for technically capable users, Rosetta is not usable for all levels of designers. Studies have pointed out the drawbacks. Firstly, back-end software that Rosetta currently support is inadequate [101]. Secondly, specialised TPL programming knowledge is required [81]. Proficiency in at least one applicable TPL is currently necessary to use Rosetta to either script GD or add new back-end; however, few designers possess TPL skills. To conclude, Rosetta can provide positive effects in programming skill learning and GD-BIM development to designers skilled in TPLs.

#### 4.3.3. Recommendations to Designers on Skill Learning and Improving

**Figure 5:**
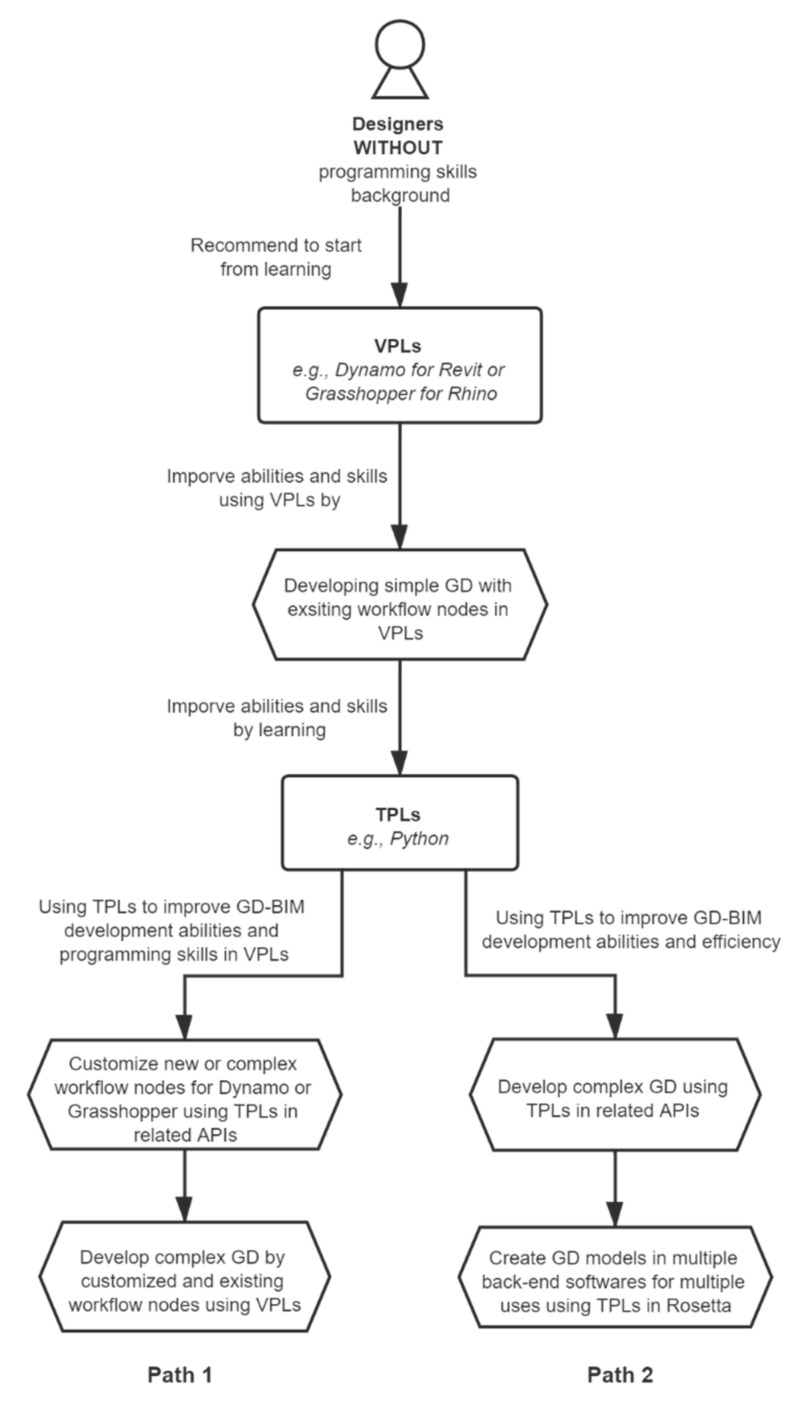
Paths of skill learning and improving GD-BIM development for designers.

Based on the analysis above, paths of skill learning and improvement of GD-BIM development are recommended to designers, as shown in Figure 5. Firstly, designers without programming skills can start by learning VPLs (e.g., Dynamo for Revit, Grasshopper for Rhino, etc.) because VPLs are intuitive and easier for novices. Secondly, after mastering a certain VPL, designers can develop simple GD with the existing workflow nodes of VPLs to improve skills. Thirdly, learning one TPL is advised as the next step because modern TPLs are more productive in developing complex tasks [17]. Python is suggested as it is easier to learn and apply compared to other complex TPLs such as C++ [80], and widely accepted by the majority of applications, including Revit and Grasshopper. Afterwards, the path can follow two directions once skilled in TPLs: (1) using TPLs to improve GD-BIM development ability and programming skills in VPLs, and (2) using TPLs and Rosetta to improve GD-BIM development ability and efficiency. Refer to the figure for detailed information. 

#### 4.3.4. Perspective of Skill-Driven GD-BIM Development

An original perspective of skill-driven GD-BIM development is proposed. Firstly, skill in at least one type of programming language (either a VPL or TPL) is necessary for designers to develop GD-BIM. Secondly, learning and proficiency in a VPL or TPL requires different investments of time and energy; thus, they are applicable to different stages and levels of GD-BIM development. Thirdly, the ability and efficiency of GD-BIM development can be progressively enhanced by strategically learning and improving VPL and TPL skills.

## 5. Discussion

There are two major aspects worth discussing based on the review and analysis. First, the GD-BIM developments to support design process have dominated the research according to the investigation of publication distribution, as shown in Figure 2. Increasingly, researchers have explored the integration of GD into BIM to establish applications or systems, allowing use by a wider group of designers. However, current attempts for this objective are still at the prototype stage and too preliminary to solve complicated design issues. Therefore, more sophisticated and systematic GD-BIM developments to support more design processes is one future research direction. 

Second, skill requirement for developing complex GD-BIM is high, because proficiency in TPLs is necessary and difficult; it requires significant time to learn and practice, which is unfriendly to most designers who are not used to programming, thus restricting wider development and application of GD-BIM. Therefore, reducing programming difficulty for designers will be another future research direction to facilitate GD-BIM development. 

As such, future directions, challenges, and potential solutions are mapped in Table 6 [109] and discussed in the following section.

### 5.1. Develop More Sophisticated and Systematic GD-BIM to Support More Design Processes

To develop and apply more sophisticated GD-BIM applications, the information below discusses challenges and methods. The current GD-BIM applications only consider limited design elements, as GD attempts to quickly resolve simple design tasks, and presents design scenes as research backgrounds too simply to facilitate prototype study. As a result, the current drawbacks are the lack of comprehensive design elements, diversity of design scenes, and mature and useful applications. Therefore, challenges are mainly three aspects: comprehensiveness of design elements, diversity of design scenes, and maturity of applications. To address these, firstly, detailed and realistic design elements such as locations, dimensions, materials, multiple types of cost, energy performance, etc. should be introduced to form complex GD constraints. Secondly, more design scenes covering diverse design processes (e.g., from conceptual design to detailed design) are advised to be studied, abstracted, and scripted into GD-BIM applications as research backgrounds. Thirdly, various complex search algorithms (e.g., genetic algorithms, shape grammars, cellular automata, etc.) can be built-in for easy selection and use, based on practical tasks to facilitate wider application of GD-BIM.

**Figure 6:**
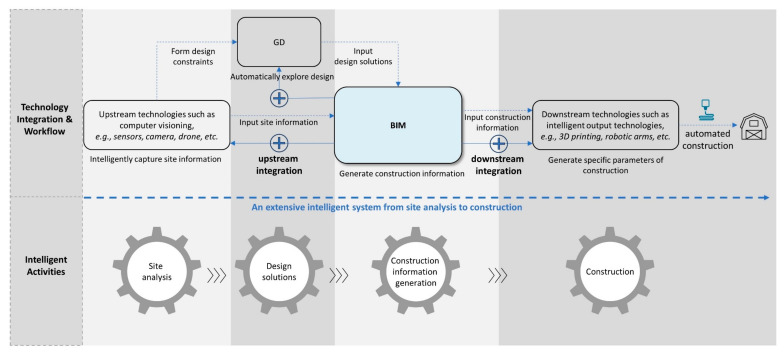
A framework of an extensive intelligent system (from site analysis to construction) formed by broad technology integration based on GD-BIM.

To implement broader integration, challenges and solutions are discussed below. The intelligence extent of GD-BIM integration has much room to expand because their current applications are mostly during the post-site analysis and pre-construction stages. Therefore, a more extensive intelligent system can largely enhance GD-BIM’s capacity to solve complicated problems. However, the largest challenge is to conduct wider integrations based on GD-BIM, to form an entire intelligent system from pre-design (site analysis) to construction. To address this, BIM can be a bridge to connect upstream and downstream technologies. A framework of an extensive intelligent system formed by broad technology integration based on GD-BIM is proposed, as presented in Figure 6. Firstly, upstream technologies such as computer visioning or sensor technology [109,110,111,112] can be integrated to intelligently capture site information and form design constraints. Secondly, downstream technologies such as 3D printing or robotic arms in construction [113,114,115,116,117] can be integrated to automatically construct the GD solutions generated beforehand. Moreover, GD-BIM demonstrates promising potential in smart engineering composites as well as artificial intelligence aided optimization [118,119,120]. Thus, an entire automated process from site analysis, design exploration, construction information generation, to design construction would be established, which could considerably save manpower and improve the buildability of designs. This broad integration grown out of GD-BIM would have great potential to solve comprehensive and complicated design issues and support multiple design processes.

### 5.2. Reduce Programming Difficulties for Designers to Facilitate GD-BIM Development

Another direction is to reduce programming difficulties for designers to enable easier development. Usually, designers in the building industry are not well equipped with programming skills, and the existing programming languages are not perfect for all level designers. Despite this, there have been instances of successful GD-BIM applications to familiarise and master programming; this is still a huge threshold for designers. Therefore, if the barriers of programming could be reduced, more designers would be willing to develop GD-BIM, which is beneficial for both technology and industry. To reduce the barriers, three potential solutions are suggested: (1) develop more compatible and portable development environments, (2) create more domain-specific and easy-to-use programming languages, and (3) establish GD library to leverage prior knowledge. 

Firstly, compatible and portable development environments, enabling designers proficient in one TPL to develop GD in multiple software for multiple usages, can reduce programming difficulty. One example of a portable development environment is Rosetta; it can reduce the requirement of learning new programming languages. However, there are shortcomings with Rosetta, such as insufficient back-end software, and high TPL skill requirements [81,101]. Thus, the current Rosetta is not usable by all levels of designers, especially those who have little TPL knowledge. To address this, more back-end connections or novice-friendly development environments accepting VPLs should be developed in the future. 

Secondly, programming languages more conforming to design languages can lower the difficulty of learning brand new languages for designers, thereby reducing programming difficulty. The text format domain-specific languages (DSLs) have this characteristic, and “modern TPLs coupling with domain-specific primitives become better alternatives to current VPLs for developing GD” [17]. They have already been applied in some GD-BIM developments for easier use of designers [57,77,84]. However, the usage of current text DSLs are still close to professional TPLs; thus, knowledge of textural programming is required. Therefore, creating more DSLs conforming to design languages and improving the availability to beginners are recommended. 

Thirdly, decreasing the reliance on programming by leveraging prior knowledge or resources can contribute to reduced programming difficulty. If the prior knowledge or resource of GD were well documented, accessed, and reused, then less programming would be required when developing new GD. For instance, three simple GD are available in Revit 2021 [63], which eliminates the demands of developing extra GD with similar functions, thus reducing programming necessity. Therefore, it inspires the recommendation of establishing expandable GD libraries in BIM that allow simple and flexible storage, access, use, and modification to decrease the reliance on programming.

## 6. Conclusions

The purpose of this review is to investigate the current approaches to developing GD in BIM by reviewing publications over the past decade. This research demonstrates a novel understanding of methodological relationships, skill requirement, and improvement for developing GD in BIM. The significance is to support designers in the building industry on the proper methods selection for developing GD-BIM. It is clear from the review and analysis that programming skills are necessary for designers to develop GD-BIM, and different types of programming languages have different suitability based on development objectives and GD components. Specifically, VPLs are more applicable to develop simple GD-BIM to solve specific practical design tasks, while TPLs are more capable of developing complex and systematic GD-BIM to establish application platforms for supporting design processes. Regarding developing GD components, TPLs are more suitable to develop abstract components such as an algorithm component or intricate design constraints, while VPLs are more suitable to develop intuitive components such as the design constraints component or simple algorithms. The skills of programming and developing can be progressively enhanced by strategically learning and improving. Accordingly, three novel perspectives of objective-oriented, GD component-based, and skill-driven GD-BIM development, as well as a set of reference guides, are proposed regarding development method selection, skill learning, and improvement.

It is also found that the GD-BIM developments to support design process have dominated the research. However, most of the current attempts are still at the prototype stage and too preliminary to solve complicated design issues. Also, the skill requirement for developing complex GD-BIM is high, as proficiency in TPLs is necessary and difficult, increasing the challenges in developing methods. Therefore, future research directions are discussed, regarding: (1) development of more sophisticated and systematic GD-BIM to support more design processes, and (2) facilitation of GD-BIM development by reducing programming difficulties for designers. The main challenges are identified, and potential solutions are recommended. 

In conclusion, this review aims to guide designers in the building industry to select proper methods or formulate skill-improving paths to develop GD-BIM and provides an inspired map for researchers to explore new knowledge.

## Figures and Tables

**Table 1:** A summary of the top five publication sources in terms of amount and year (2010–2020).

Source Title	2010	2011	2012	2013	2014	2015	2016	2017	2018	2019	2020	Sum
CAADRIA		1	1	1	2	1	2	3		2		13
Automation in Construction				2		1			4	2	1	10
IOP Conference Series: Earth and Environmental Science										3	3	6
ISARC				1			3	1	1			6
Procedia Engineering					2		2					4
Sum	0	1	1	4	4	2	7	4	5	7	4	39

**Table 3:** Comparison between VPLs and TPLs regarding definitions, languages, advantages, and limitations.

Categories	Definitions	Languages	Advantages	Limitations
Visual programming languages (VPLs)	Any programming language that allows users to develop programs by manipulating visual elements interactively.	Grasshopper Dynamo Optimizer Node etc.	Simpler operation. Intuitive and interactive. Immediate visual feedback. Good Interactive Development Environment (IDE). Processes can be described graphically and intuitively by a form of ‘pipes-and-filters’ logic. Convenient for small program development. Faster learning curve. Little programming background requirement. More productive and motivating for novices.	Apply to very limited domains and highly specialized solutions. Scale poorly with large and complex design tasks. Absence of (sophisticated) abstraction mechanisms, resulting in redundancy.
Textural programming languages (TPLs)	Any programming language that uses lines of text, code, symbol, predefined syntax, etc. to develop programs.	Python C# C++ JavaScript AutoLISP Haskell VisualScheme etc.	Various types of abstraction mechanisms. Easy to adjust to changing requirements. Significantly more productive for large scale and complex design tasks.	Absence of good IDE. Hard to learn for novices. Steeper learning curve. Require extensive knowledge and proficient skill.

**Table 4:** Commonly used software and applicable programming languages for scripting GD.

Software	Developer	BIM	Connectable to BIM	Plug-In or Stand-Alone	Applicable Programming Languages for Scripting GD	
					VPLs	TPLs
Revit	Autodesk	Yes	N/A	Stand-alone	Dynamo	Python, C# in Revit API
ArchiCAD	Graphisoft	Yes	N/A	Stand-alone	Grasshopper—Archicad Live Connection	C++ in ArchiCAD API
Grasshopper	McNeel	No	Yes; by Lyrebird, etc.	Plug-in for Rhinoceros	Grasshopper	GhPython in Grasshopper API
GenerativeComponents	Bentley	No	Yes. GenerativeComponents CONNECT Edition.	Stand-alone and Plug-in for MicroStation	Optimizer Node in CONNECTION Edition	GCScript, C#

**Table 5:** Suitability relationship between programming languages and GD components development.

Programming Languages	Characteristics of Programming Languages on Fundamental Dimensions			Suitability for GD Component Development
	Primitive	Combination	Abstraction	
VPLs Such as: Dynamo Grasshopper Optimizer Node	Implement a large set of primitive components (e.g., ranges, mappings, and geometric operations). Primitive components are with a high degree of sophistication. Good in expressing intuitive ideas by connecting primitives.	Provide a single combination paradigm. Rely on an extremely simple metaphor (primitives combined by connecting outputs and inputs of components). This metaphor is too restrictive to express: ➢ complex control structure (e.g., iteration, recursion, etc.), and ➢ algorithms without using a TPL to script customized primitive components. Scale poorly with complex design tasks, may result in reading and maintenance issues.	Provide ‘Cluster’ function, which allows to group other components or clusters as a single component. Good for improving programs clarity and parts reuse. Inability in centralized definition (as clusters are independent from their copies). Not real abstraction.	Intuitive design constraints, or straightforward algorithms.
TPLs Such as: Python C# C++ JavaScript AutoLISP Haskell VisualScheme	Currently not as many primitives as VPLs.	Provide various combination paradigms, such as functional, imperative, and object-oriented. Including expression composition, and various control and data structures. Paradigms are extendable. Relay on underlying languages. Expressive in complex design tasks.	Provide various types of procedurals, data, and control abstraction. Good in simplifying solutions. Much easier to adjust to changing requirements. Significantly more abstract than VPLs.	Abstract algorithms, or intricate design constraints.

**Table 6:** Future directions of GD-BIM development and corresponding challenges and potential solutions.

Future Directions	Challenges	Potential Solutions
Develop more sophisticated and systematic GD-BIM to support more design processes.	Lack of diversity of GD design scenes. Lack of consideration for comprehensive design elements. Lack of mature GD-BIM environments convenient to use for complicated design. Lack of wider integration to form a more extensive intelligent system. High development skill requirement.	Explore more sophisticated design scenes. Introduce detailed and realistic design elements as GD constraints. Built-in complex search algorithms for convenient selection and use. Develop more industry-usable GD-BIM applications for complex design. Integrate with upstream or downstream technologies such as computer visioning or intelligent output.
Reduce programming difficulties for designers.	High programming requirement for designers. Lack of prior knowledge and resource of GD.	Develop more diversified, and novice-friendly, compatible, and portable development environments. Create more domain-specific and easy-to-use programming languages. Establish expandable GD library to leverage prior knowledge.
