# Correction: Ma et al. Generative Design in Building Information Modelling (BIM): Approaches and Requirements. *Sensors* 2021, *21*, 5439

**DOI:** 10.3390/s26041261

**Published:** 2026-02-14

**Authors:** Wei Ma, Xiangyu Wang, Jun Wang, Xiaolei Xiang, Junbo Sun

**Affiliations:** 1 School of Design and the Built Environment, Curtin University, Bentley, WA 6102, Australia; wei.ma@curtin.edu.au (W.M.); xiaolei.xiang@curtin.edu.au (X.X.); tunneltc@gmail.com (J.S.); 2 School of Civil Engineering and Architecture, East China Jiao Tong University, Nanchang 330013, China; 3 Australasian Joint Research Centre for Building Information Modelling, School of Built Environment, Curtin University, Perth, WA 6102, Australia; 4 School of Engineering, Design and Built Environment, Western Sydney University, Kingswood, NSW 2747, Australia; jun.wang@westernsydney.edu.au

Following publication, concerns were raised regarding the relevance of a few references in this publication [1]. Adhering to our standard procedure, the Editorial Board conducted an investigation which determined that the original references [21,114,115,122–124,128] are irrelevant to the content of the article. Additionally, the original reference [121] has been retracted. As a result, following discussion with the authors, the Editorial Board has decided to remove the references [21,114,115,121–124,128] from this publication [1]. With this correction, the order of some references has been adjusted accordingly.

The first author Wei Ma’s e-mail address has been updated to <wei.ma@curtin.edu.au>.

The authors state that the scientific conclusions are unaffected. This correction was approved by the Academic Editor. The original publication has also been updated.
